# Causes of ischemic stroke in young adults versus non-young adults: A multicenter hospital-based observational study

**DOI:** 10.1371/journal.pone.0268481

**Published:** 2022-07-13

**Authors:** Yuichiro Ohya, Ryu Matsuo, Noriko Sato, Fumi Irie, Kuniyuki Nakamura, Yoshinobu Wakisaka, Tetsuro Ago, Masahiro Kamouchi, Takanari Kitazono, Investigators for Fukuoka Stroke Registry

**Affiliations:** 1 Department of Medicine and Clinical Science, Graduate School of Medical Sciences, Kyushu University, Fukuoka, Japan; 2 Department of Health Care Administration and Management, Graduate School of Medical Sciences, Kyushu University, Fukuoka, Japan; 3 Center for Cohort Studies, Graduate School of Medical Sciences, Kyushu University, Fukuoka, Japan; National Cerebral And Cardiovascular Center, JAPAN

## Abstract

**Background:**

Very few comparative studies have focused on the differences in the causes of ischemic stroke between young adults and non-young adults. This study was performed to determine what causes of ischemic stroke are more important in young adults than in non-young adults using a large-scale multicenter hospital-based stroke registry in Fukuoka, Japan.

**Methods and results:**

We investigated data on 15,860 consecutive patients aged ≥18 years with acute ischemic stroke (mean age: 73.5 ± 12.4 years, 58.2% men) who were hospitalized between 2007 and 2019. In total, 779 patients were categorized as young adults (≤50 years of age). Although vascular risk factors, including hypertension, diabetes mellitus, and dyslipidemia, were less frequent in young adults than in non-young adults, the prevalence of diabetes mellitus and dyslipidemia in young adults aged >40 years were comparable to those of non-young adults. Lifestyle-related risk factors such as smoking, drinking, and obesity were more frequent in young adults than in non-young adults. As young adults became older, the proportions of cardioembolism and stroke of other determined etiologies decreased, but those of large-artery atherosclerosis and small-vessel occlusion increased. Some embolic sources (high-risk sources: arterial myxoma, dilated cardiomyopathy, and intracardiac thrombus; medium-risk sources: atrial septal defect, nonbacterial thrombotic endocarditis, patent foramen ovale, and left ventricular hypokinesis) and uncommon causes (vascular diseases: reversible cerebral vasoconstriction syndrome, moyamoya disease, other vascular causes, arterial dissection, and cerebral venous thrombosis; hematologic diseases: antiphospholipid syndrome and protein S deficiency) were more prevalent in young adults than in non-young adults, and these trends decreased with age.

**Conclusions:**

Certain embolic sources and uncommon causes may be etiologically important causes of ischemic stroke in young adults. However, the contribution of conventional vascular risk factors and lifestyle-related risk factors is not negligible with advancing age, even in young adults.

## Introduction

Stroke is most common in aged people; however, a certain percentage of stroke may occur in young adults. Recent studies have estimated that the rate of ischemic stroke in young adults ranges from 15% to 18% of ischemic strokes [1, 2]. The incidence of ischemic stroke affecting young adults reportedly ranges from 7 to >100 per 100,000 person-years depending on the country [3]. Because young adults are expected to live with their disability for a longer time after stroke, the impact of stroke on quality of life is more serious in young adults than in older patients. Given this background, prevention of stroke in young adults is an imminent public health issue worldwide.

Causes of ischemic stroke in young adults are presumed to differ from those in non-young adults. Many studies to date have investigated clinical data in the population of young adult patients with stroke. However, the age cut-off for young adults in these studies varied from 40 to 60 years old, while the findings among the studies highly differ with respect to risk factors, stroke subtypes, and causes of ischemic stroke in young adults [4–29]. Additionally, few studies have compared causes of ischemic stroke between young adults and non-young adults. Consequently, the differences in the causes of ischemic stroke between young adults and non-young adults remain ambiguous.

This study was performed to elucidate how vascular risk factors, stroke etiology, and particularly causes of ischemic stroke differ between young adults and non-young adults. To this end, we investigated clinical data in consecutive patients with acute ischemic stroke aged ≥18 years who were enrolled in a large-scale, multicenter, hospital-based cohort study of stroke in Fukuoka, Japan and compared these data between young adults and non-young adults.

## Materials and methods

### Study design

We constructed a multicenter hospital-based stroke registry, the Fukuoka Stroke Registry, in Fukuoka, Japan (UMIN Clinical Trials Registry: UMIN-CTR 0000008000). The Fukuoka Stroke Registry enrolled consecutive patients with acute stroke who were hospitalized within 7 days of onset in seven participating stroke centers in Fukuoka, Japan. The participating hospitals were Kyushu University Hospital (Fukuoka, Japan), National Hospital Organization Kyushu Medical Center (Fukuoka, Japan), National Hospital Organization Fukuoka–Higashi Medical Center (Koga, Japan), Fukuoka Red Cross Hospital (Fukuoka, Japan), St. Mary’s Hospital (Kurume, Japan), Steel Memorial Yawata Hospital (Kitakyushu, Japan), and Japan Labour Health and Welfare Organization Kyushu Rosai Hospital (Kitakyushu, Japan). The institutional review committees of all hospitals approved the study protocol. We obtained written informed consent on admission from all eligible participants or their family members for the follow-up study. For the secondary analysis of anonymized hospitalization data, the institutional review boards waived the requirement for written informed consent by providing patients and their family members with an opportunity to opt out. Stroke was defined as sudden onset of a nonconvulsive and focal neurological deficit, and ischemic stroke was diagnosed using brain computed tomography (CT), magnetic resonance (MR) imaging, or both.

The study data, methods used in the analysis, and materials used to conduct the research will be made available to any researcher for purposes of reproducing the results or replicating the procedure. The data that support the findings of this study are available from the Fukuoka Stroke Registry at Kyushu University upon reasonable request.

### Study patients

We scrutinized data of all consecutive patients with acute ischemic stroke who were hospitalized in the participating hospitals of the Fukuoka Stroke Registry. Of 18,166 patients with acute ischemic stroke who were hospitalized from June 2007 to September 2019, we excluded 2,296 patients who did not receive reperfusion therapy but had a focal neurological deficit lasting <24 hours, because definite causes were often not identified when neurological symptoms spontaneously disappeared within a short time. However, patients who received reperfusion therapy were included regardless of neuroimaging findings, even when neurological deficits were rapidly recovered by therapy. After additionally excluding 10 patients aged <18 years, a total of 15,860 patients were included in the final analysis (S1 Fig). The study patients were then categorized into two groups according to age: young adult patients aged 18 to 50 years and non-young adult patients aged >50 years [30].

### Risk factors

We investigated the patients’ background characteristics, including demographics, history of stroke, conventional vascular risk factors, and lifestyle-related risk factors. A history of stroke was defined as a history of ischemic or hemorrhagic stroke before hospitalization for the index ischemic stroke. Conventional vascular risk factors included hypertension, diabetes mellitus, and dyslipidemia. Lifestyle-related risk factors included smoking habit, drinking habit, and obesity. The definitions of risk factors in this study were as follows: hypertension (systolic blood pressure of ≥140 mmHg or diastolic blood pressure of ≥90 mmHg in the chronic stage or pre-stroke treatment with antihypertensive medication), diabetes mellitus (fasting blood glucose concentration of ≥7.0 mmol/L, casual blood glucose concentration of ≥11.1 mmol/L, positive 75-g oral glucose tolerance test result, or glycated hemoglobin A1c concentration of ≥6.5% on 2 different days during hospitalization or in the chronic stage, or pre-stroke treatment with antidiabetic medication), dyslipidemia (either a low-density lipoprotein-cholesterol level of 3.62 mmol/L, high-density lipoprotein-cholesterol level of 1.03 mmol/L, triglyceride level of 1.69 mmol/L, or pre-stroke treatment with a cholesterol-lowering drug), smoking (previous or current cigarette smoking), drinking (habitual consumption of alcoholic beverages), and obesity (body mass index ≥25). Body mass index was calculated using the body weight and height measured at admission.

### Stroke subtype

Stroke subtypes were classified based on the Trial of ORG 10172 in Acute Stroke Treatment (TOAST) criteria [31]. The TOAST classification system includes five categories: large-artery atherosclerosis, small-vessel occlusion, cardioembolism, other determined etiology, and undetermined etiology. The diagnostic flow chart that was used to determine stroke etiology in this study is shown in S2 Fig.

The definitions of stroke subtypes in this study were as follows. Cardioembolism was defined as an embolism of cardiac origin based on the TOAST criteria and a territorial infarct, cortical branch infarct, or large infarct in the territory of multiple perforating arteries without an occlusive lesion in the proximal arteries. High- and medium-risk embolic sources were regarded as causes of cardioembolism. Large-artery atherosclerosis was diagnosed by an occlusive lesion (significant stenosis [>50%], ulceration, or occlusion) due to atherosclerosis in the proximal arteries, and a border zone infarct, cortical branch infarct, or large infarct in the territory of multiple perforating arteries without other embolic sources. Small-vessel occlusion was diagnosed based on clinical symptoms compatible with lacunar syndrome, and a small infarct in the territory of the perforating artery without occlusive arterial lesion. Other determined etiology was considered present when the stroke subtype fulfilled neither cardioembolism, large-artery atherosclerosis, nor small-vessel occlusion and other potential causes were identified.

### Embolic sources of cardioembolism

Embolic sources of cardioembolism were classified into high- and medium-risk sources based on the TOAST criteria [31]. When multiple embolic sources were found in one patient, all of them were listed. High- and medium-risk embolic sources were categorized based on the TOAST criteria. Atrial fibrillation was diagnosed based on electrocardiographic findings on admission or during hospitalization or a history of atrial fibrillation. Atrial fibrillation with mitral stenosis, atrial fibrillation with other valvular diseases, and atrial fibrillation without any valvular disease were grouped into atrial fibrillation irrespective of its duration (paroxysmal, persistent, and permanent atrial fibrillation). Recent and old myocardial infarction was defined as myocardial infarction within 4 weeks of onset and that between 4 weeks and 6 months of onset, respectively. Patent foramen ovale was diagnosed when microbubbles were found passing through the foramen ovale from the right to left atrium during the Valsalva maneuver after intravenous injection of a contrast agent [32, 33]. Nonbacterial thrombotic endocarditis was defined as vegetations detected by echocardiography in patients with advanced cancer in the absence of systemic infection [32, 33]. Embolic sources were grouped into a prosthetic cardiac valve when we did not know whether patients had a bioprosthetic or mechanical valve. Intracardiac thrombus included the left atrial/atrial appendage and ventricular thrombus. Left ventricular akinesis and hypokinesis were listed as potential embolic sources when an akinetic and hypokinetic left ventricular segment was considered as having caused the embolism, respectively. Although atrial septal defect is not listed in the TOAST criteria, it was regarded as a medium-risk embolic source of cardioembolism in this study. Embolic sources were diagnosed primarily based on the Japanese Circulation Society guidelines.

### Uncommon causes as other determined etiologies

Other potential causes were termed “uncommon causes.” Uncommon causes were classified into three groups according to their origins: vascular diseases, hematologic diseases, and other miscellaneous diseases. Potential embolic sources that were not listed in the TOAST criteria were categorized as uncommon causes. When more than one uncommon cause was identified in one patient, all of them were also listed.

Uncommon causes were classified into vascular diseases (arterial dissection, moyamoya disease, aortic arch atherosclerotic plaque, cerebral venous thrombosis, reversible cerebral vasoconstriction syndrome, angiitis, arteriogenic embolism, aortic dissection, and other vascular causes), hematologic diseases (antiphospholipid antibody syndrome, protein S deficiency, hypereosinophilic syndrome, protein C deficiency, anemia, essential thrombocythemia, polycythemia, and other coagulopathies), and miscellaneous causes (hyperhomocysteinemia, drugs, post-catheter, pulmonary arteriovenous fistula, and miscellaneous causes). These uncommon causes were diagnosed based on the diagnostic criteria in the clinical practice guidelines specific to each disease.

Reversible cerebral vasoconstriction syndrome included putative precipitants and associated disorders, such as vasoactive drugs (e.g., illicit drugs), post-partum (e.g., eclampsia), and posterior reversible encephalopathy syndrome, as well as migraine [34]. Other vascular causes included potential causes of vascular origin, such as quasi-moyamoya disease, mechanical compression of an artery, aortitis syndrome, carotid web, an operative procedure, and hypoplasia of the internal carotid artery. Arteriogenic embolism included embolism from proximal arteries such as a mural thrombus of an aortic aneurysm or stent, embolism from a thrombus of a cerebral aneurysm, arteriogenic embolism from a non-stenotic carotid artery and subclavian artery plaques with ulcerated or irregular surfaces, embolism from thrombosis in the pulmonary vein stump, and cholesterol embolism. Aortic arch atherosclerotic plaques were defined as aortic arch atheroma with complex aortic plaques identified by transesophageal echocardiography or CT angiography. Complex aortic plaques included plaques of ≥4-mm thickness, ulceration, or mobile plaques [32, 33]. Other coagulopathies included disseminated intravascular coagulation, hyperviscosity syndrome secondary to hematologic diseases, chronic inflammation, and antithrombin III deficiency as a cause of ischemic stroke. Types of anemia that were regarded as a cause of ischemic stroke in this study included iron deficiency anemia, anemia due to bleeding, anemia of inflammation, renal anemia, and hematopoietic diseases. Drugs that were considered causes of ischemic stroke in this study included oral contraceptive/post-menopausal hormone therapy, antineoplastic agents, thrombopoietin-stimulating agents, and steroids. Miscellaneous causes included sarcoidosis, pregnancy-related causes, intravascular lymphoma, and hypotensive shock.

### Statistical analysis

Background characteristics were compared between young adults and non-young adults by the two-tailed t test, Mann–Whitney U test, Pearson’s chi-squared test, or Fisher’s exact test as appropriate. Trends in the vascular risk factors, stroke subtypes, embolic sources, and uncommon causes according to age categories were analyzed by the Cochran–Armitage test. Multiple comparisons were performed between young adults of each age category and non-young adults by Tukey’s wholly significant difference test. Odds ratios and 95% confidence intervals of the causes of ischemic stroke were estimated for young adults in reference to non-young adults by logistic regression analysis. Sex differences in the frequencies of embolic sources or uncommon causes in young adults were tested by the Fisher’s exact test. All analyses were performed using Stata 16.0 software (StataCorp, College Station, TX, USA). All tests were two-sided, and a P value of <0.05 was considered statistically significant.

## Results

### Risk factors

The mean ± standard deviation age of the 15,860 patients was 73.5 ± 12.4 years, and 58.2% were men. Of all patients, 779 were young adults (mean age: 43.0 ± 6.7 years, 66.0% men) (S3 Fig).

The proportion of men was significantly higher among young adults than non-young adults (Table 1). The prevalence of hypertension, diabetes mellitus, dyslipidemia, and a history of stroke were significantly lower in young adults than in non-young adults. By contrast, smoking, drinking, and obesity were more frequent in young adults than in non-young adults.

**Table 1:** Background characteristics of young adults and non-young adults.

	Young adults n = 779	Non-young adults n = 15081	P
Age, years	43.0 ± 6.7	75.1 ± 10.5	<0.001
Men	514 (66.0)	8715 (57.8)	<0.001
Vascular risk factors			
Hypertension	452 (58.0)	12331 (81.8)	<0.001
Diabetes mellitus	184 (23.6)	4822 (32.0)	<0.001
Dyslipidemia	373 (47.9)	7935 (52.6)	0.01
Lifestyle-related factors			
Smoking	500 (64.2)	7530 (49.9)	<0.001
Drinking	327 (42.0)	4811 (31.9)	<0.001
Obesity	343 (44.3)	3511 (23.6)	<0.001
Previous stroke	67 (8.6)	3452 (22.9)	<0.001

Data are presented as mean ± standard deviation or n (%).

The prevalence of vascular risk factors and lifestyle-related risk factors were further evaluated according to 5-year age increments and compared between young adults and non-young adults (S1 Table). The prevalence of hypertension, diabetes mellitus, dyslipidemia, smoking, and drinking increased with 5-year age increments, even among young adults.

**Fig 1:**
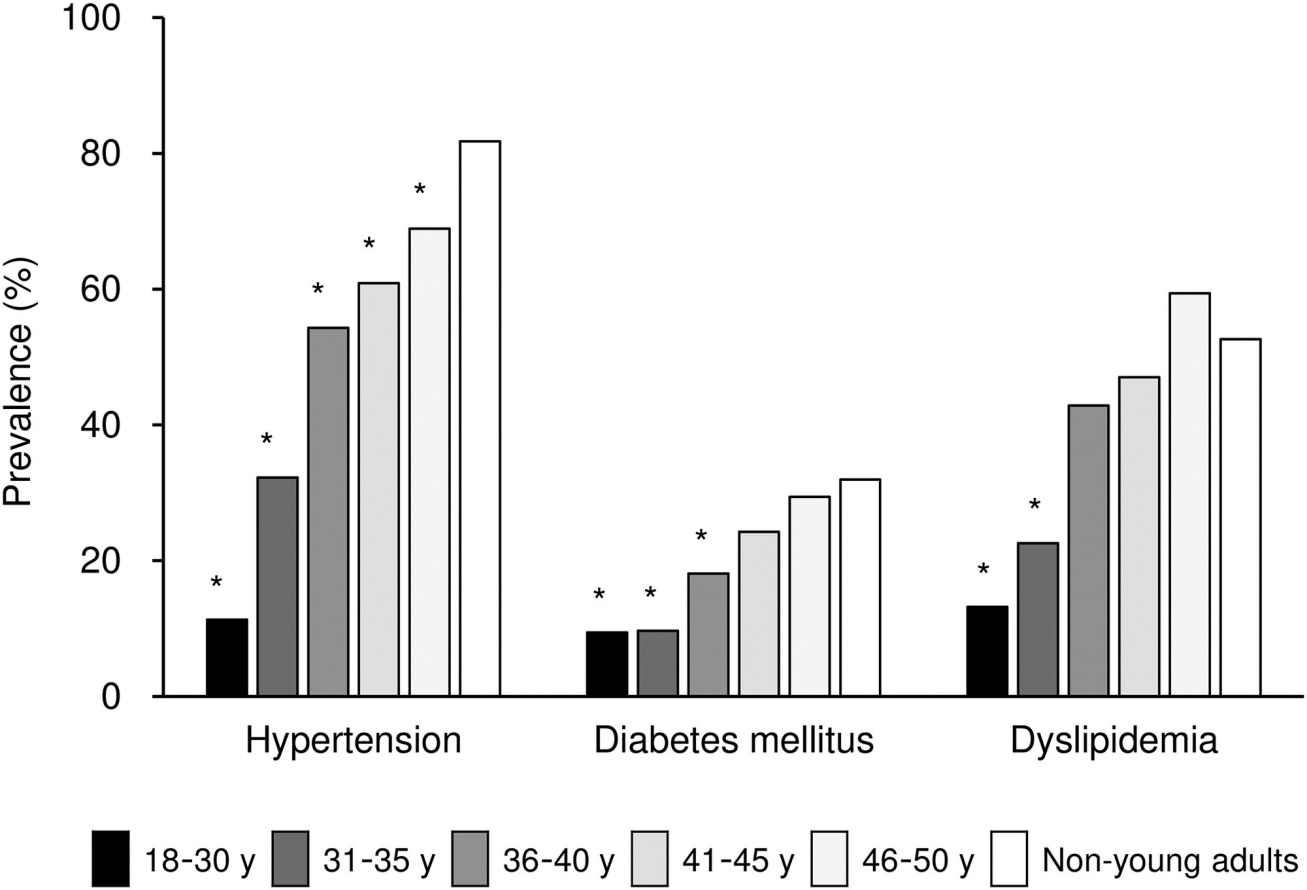
Prevalence of vascular risk factors according to age among young adults versus non-young adults. Prevalence of hypertension, diabetes mellitus, dyslipidemia, and smoking is shown in patients of each age category (18–30 years, 31–35 years, 36–40 years, 41–45 years, 46–50 years, and non-young adults [>50 years]). *P < 0.05 vs. non-young adults by multiple comparison.

**Fig 2:**
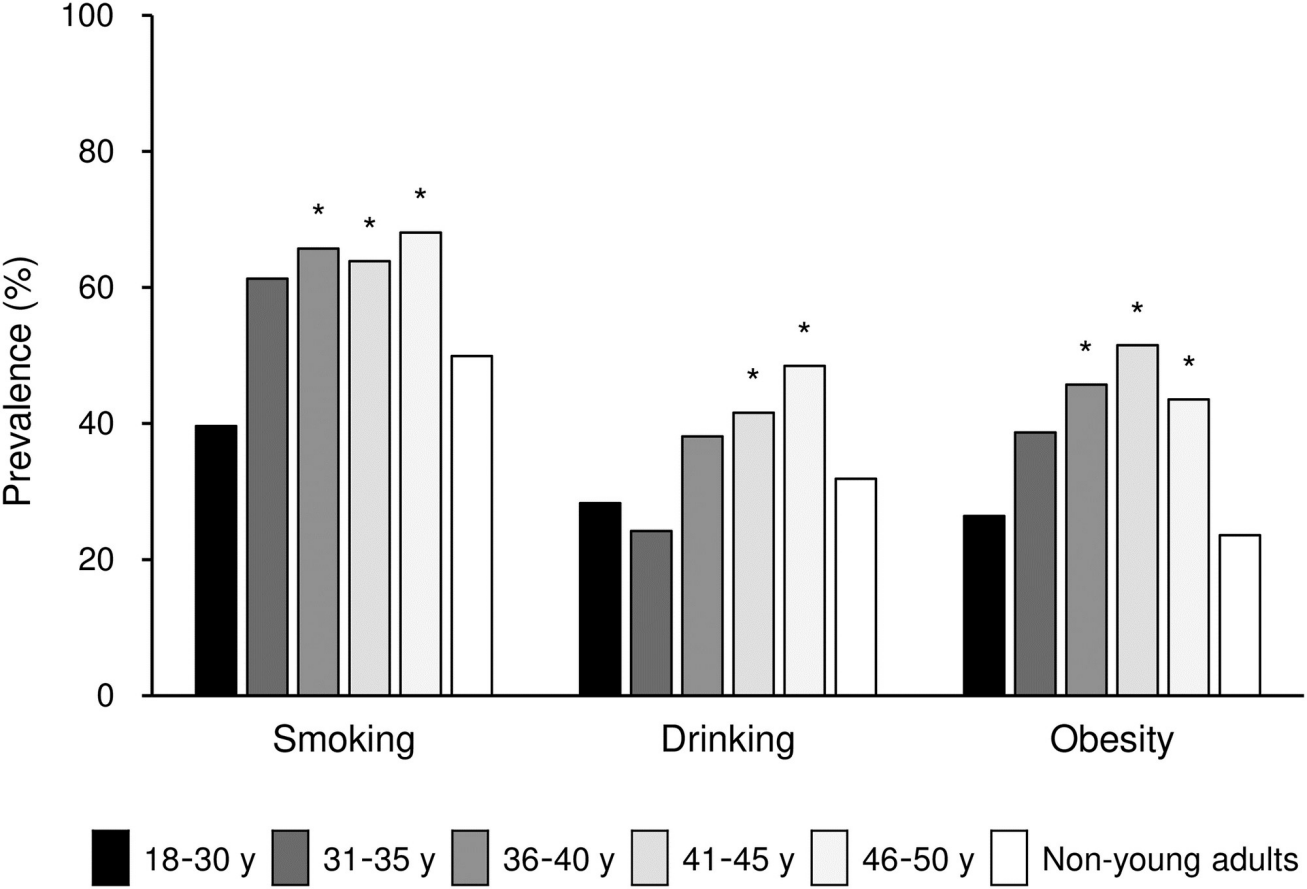
Prevalence of lifestyle-related risk factors according to age among young adults versus non-young adults. Prevalence of smoking, drinking, and obesity is shown in patients of each age category (18–30 years, 31–35 years, 36–40 years, 41–45 years, 46–50 years, and non-young adults [>50 years]). *P < 0.05 vs. non-young adults by multiple comparison.

Multiple comparison revealed that the prevalence of diabetes mellitus in patients aged >40 years and dyslipidemia in patients aged >35 years were not significantly different from those in non-young adults (Fig 1, S1 Table). By contrast, smoking habit and obesity were more frequent in young adults aged >35 years than in non-young adults (Fig 2, S1 Table). Similarly, drinking habit was more frequent in young adults aged >40 years than in non-young adults (Fig 2, S1 Table).

### Stroke subtype

Stroke subtypes significantly differed between young adults and non-young adults (Table 2). Cardioembolism and large-artery atherosclerosis were less frequent whereas other determined etiologies and undetermined etiologies were more frequent in young adults than in non-young adults. Among the potential causes of ischemic stroke, high-risk embolic sources were predominant in non-young adults, whereas medium-risk sources accounted for a substantial part of embolic sources in young adults.

**Table 2:** Stroke subtype of young adults and non-young adults.

	Young adults n = 779	Non-young adults n = 15081	P
Cardioembolism	49 (6.3)	3883 (25.8)	<0.001
High-risk sources	31 (4.0)	3653 (24.2)	
Medium-risk sources	18 (2.3)	230 (1.5)	
Large-artery atherosclerosis	82 (10.5)	2627 (17.4)	<0.001
Small-vessel occlusion	240 (30.8)	4521 (30.0)	0.62
Other determined etiology	207 (26.6)	1278 (8.5)	<0.001
Undetermined cause	201 (25.8)	2772 (18.4)	<0.001

Data are presented as n (%).

**Fig 3:**
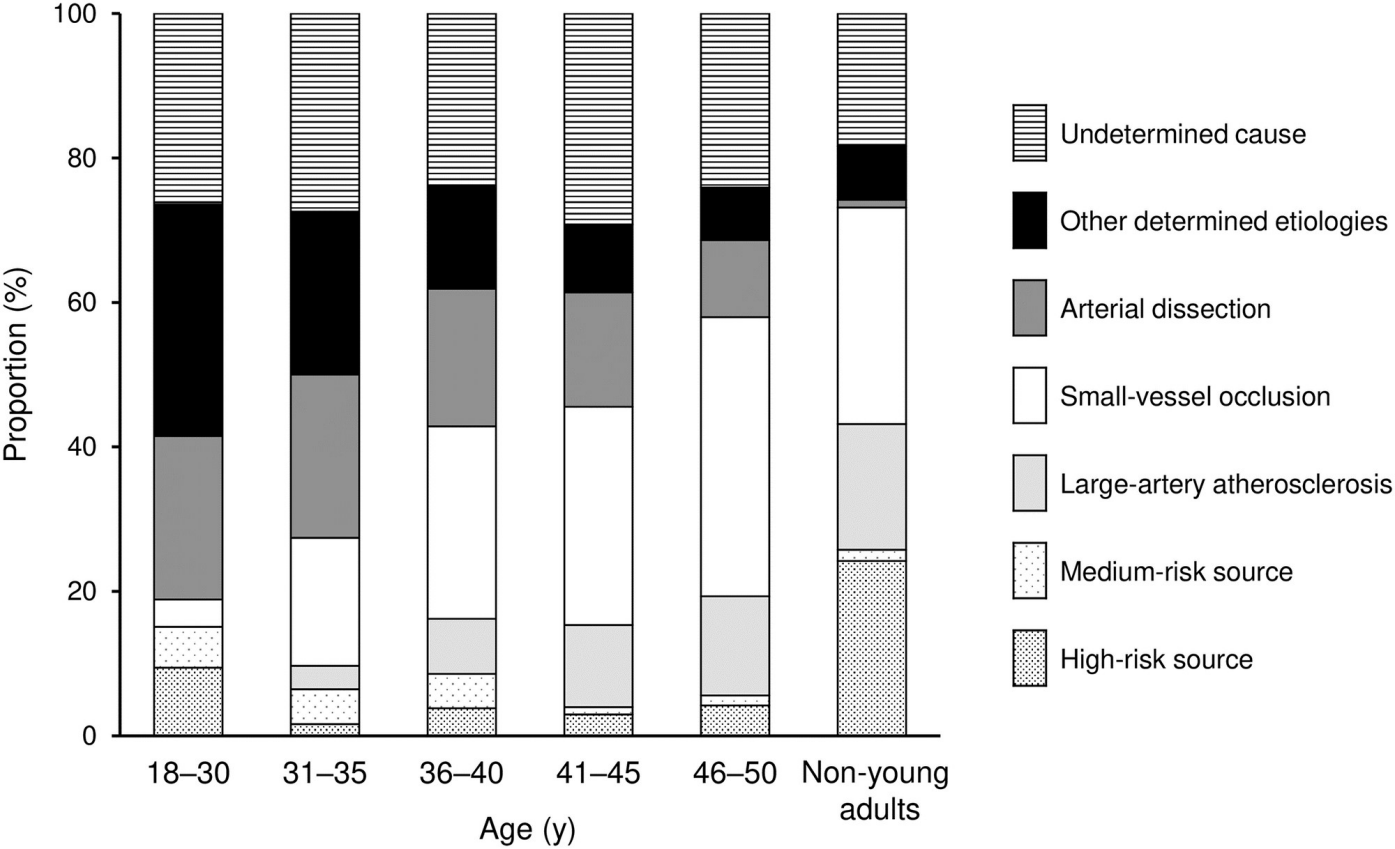
Proportion of stroke subtypes according to age among young adults versus non-young adults. Proportions of cardioembolism (high-risk sources and medium-risk sources), large-artery atherosclerosis, small-vessel occlusion, other determined cause (arterial dissection and others), and undetermined cause are shown in patients of each age category (18–30 years, 31–35 years, 36–40 years, 41–45 years, 46–50 years, and non-young adults [>50 years]).

Among young adult patients, the proportions of cardioembolism and other determined etiologies decreased with 5-year age increments, whereas small-vessel occlusion and large-artery atherosclerosis increased with age (Fig 3, S2 Table).

### Embolic sources of cardioembolism

Various diagnostic assessments were performed to determine the etiology of ischemic stroke and possible causes of ischemic stroke in individual patients. The frequencies of 24-hour ambulatory Holter monitoring and transthoracic echocardiography were similar between young adults and non-young adults (Table 3). However, echocardiography, especially transesophageal echocardiography, was performed more frequently in young adults compared with non-young adults, especially in patients with cardioembolic stroke.

**Table 3:** Cardiac assessments for stroke etiology in young adults and non-young adults.

	Young adults	Non-young adults	P
All subtypes	n = 779	n = 15081	
12-lead electrocardiography	779 (100.0)	15081 (100.0)	1.00
24-hour ambulatory Holter monitoring	651 (83.6)	12419 (82.4)	0.38
Transthoracic echocardiography	736 (94.5)	14190 (94.1)	0.65
Transesophageal echocardiography	238 (30.6)	2838 (18.8)	<0.001
Cardioembolism	n = 49	n = 3883	
12-lead electrocardiography	49 (100.0)	3883 (100.0)	1.00
24-hour ambulatory Holter monitoring	37 (75.5)	2645 (68.1)	0.27
Transthoracic echocardiography	49 (100.0)	3584 (92.3)	0.04
Transesophageal echocardiography	25 (51.0)	602 (15.5)	<0.001

Data are presented as n (%).

**Fig 4:**
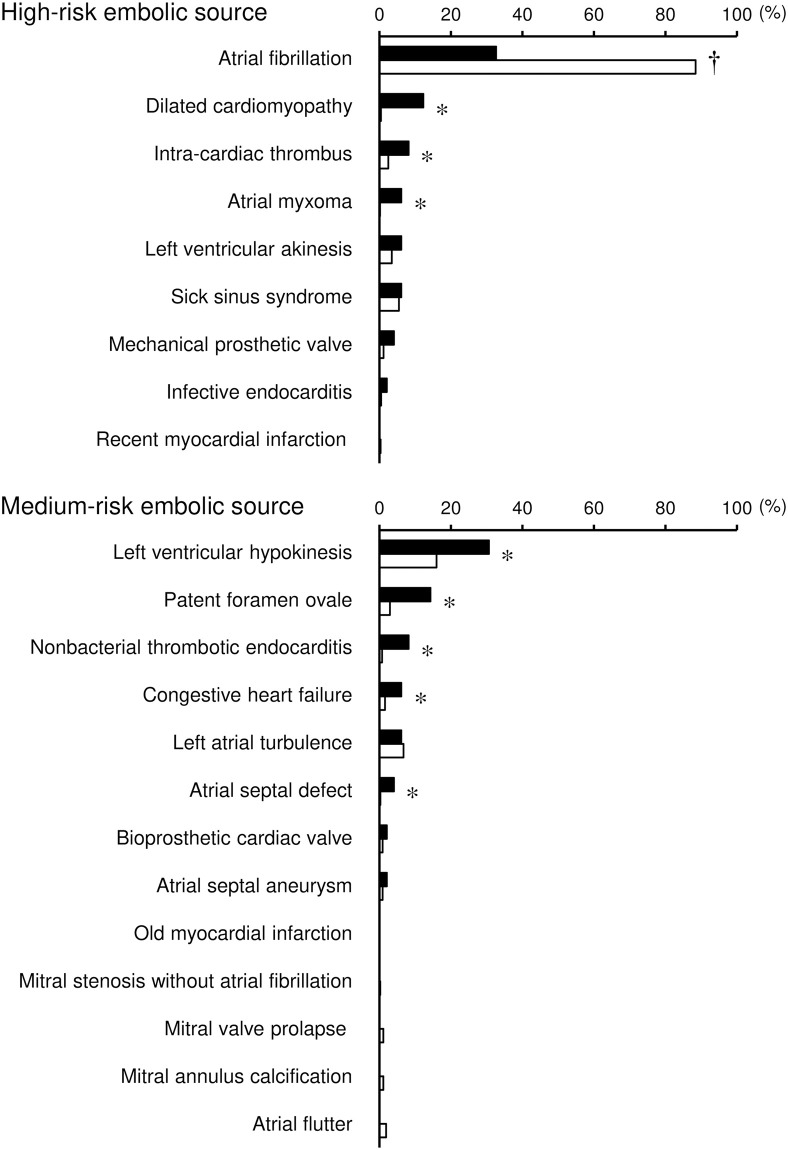
Frequency of embolic sources in young adults and non-young adults. Frequency of embolic sources in young adults (closed columns) and non-young adults (open columns) is shown according to risk stratification (high- and medium-risk embolic sources) among patients with ischemic stroke due to cardioembolism. When patients had multiple embolic sources, all potential sources were listed. *P < 0.05, higher in young adults than in non-young adults. †P < 0.05, lower in young adults than in non-young adults.

Based on these cardiac assessment findings, a variety of embolic sources were found as potential causes of cardioembolism in the total group of patients (Fig 4, S3 Table). The frequencies of medium-risk sources of cardioembolism decreased with increasing age, even in young adults, although there were no age-dependent changes for high-risk sources in young adults (Fig 3, S2 Table). Three high-risk embolic sources (dilated cardiomyopathy, intracardiac thrombus, and arterial myxoma) and five medium-risk embolic sources (left ventricular hypokinesis, patent foramen ovale, nonbacterial thrombotic endocarditis, congestive heart failure, and atrial septal defect) were significantly more frequent in young adults than in non-young adults. By contrast, atrial fibrillation was less frequent in young adults than in non-young adults.

To further confirm age-dependent trends in the frequency of these embolic sources, we evaluated whether the 10-year trends were statistically significant among the overall patients (S4 Fig, S3 Table). With the exception of congestive heart failure, all embolic sources that were more frequent in young adults than in non-young adults showed significantly decreasing trends according to 10-year age increments. Conversely, the frequency of atrial fibrillation remarkably increased with advancing age.

### Uncommon causes as other determined etiologies

Almost all patients underwent brain MR imaging, although the frequency of MR imaging use was slightly higher in young adults than in non-young adults (Table 4). The frequency of carotid ultrasonography was similar between young adults and non-young adults. Extracranial and intracranial arteries were more frequently assessed by CT angiography, MR angiography, and conventional angiography in young adults than in non-young adults. In patients with ischemic stroke due to other determined etiologies, the frequencies of CT angiography and conventional angiography were higher in non-young adults than in young adults.

**Table 4:** Vascular assessments for stroke etiology in young adults and non-young adults.

	Young adults	Non-young adults	P
All subtypes	n = 779	n = 15081	
Brain CT	552 (70.9)	11417 (75.7)	0.002
Brain MR imaging	770 (98.8)	14674 (97.3)	0.009
Carotid ultrasonography	719 (92.3)	13951 (92.5)	0.83
CT angiography	244 (31.3)	2385 (15.8)	<0.001
MR angiography	766 (98.3)	14601 (96.8)	0.02
Conventional angiography	113 (14.5)	1032 (6.8)	<0.001
Other determined etiologies	n = 207	n = 1278	
Brain CT	155 (74.9)	863 (67.5)	0.04
Brain MR imaging	206 (99.5)	1257 (98.4)	0.20
Carotid ultrasonography	189 (91.3)	1223 (95.7)	0.007
CT angiography	115 (55.6)	298 (23.3)	<0.001
MR angiography	205 (99.0)	1252 (98.0)	0.30
Conventional angiography	59 (28.5)	93 (7.3)	<0.001

Data are presented as n (%).

CT: computed tomography, MR: magnetic resonance.

**Fig 5:**
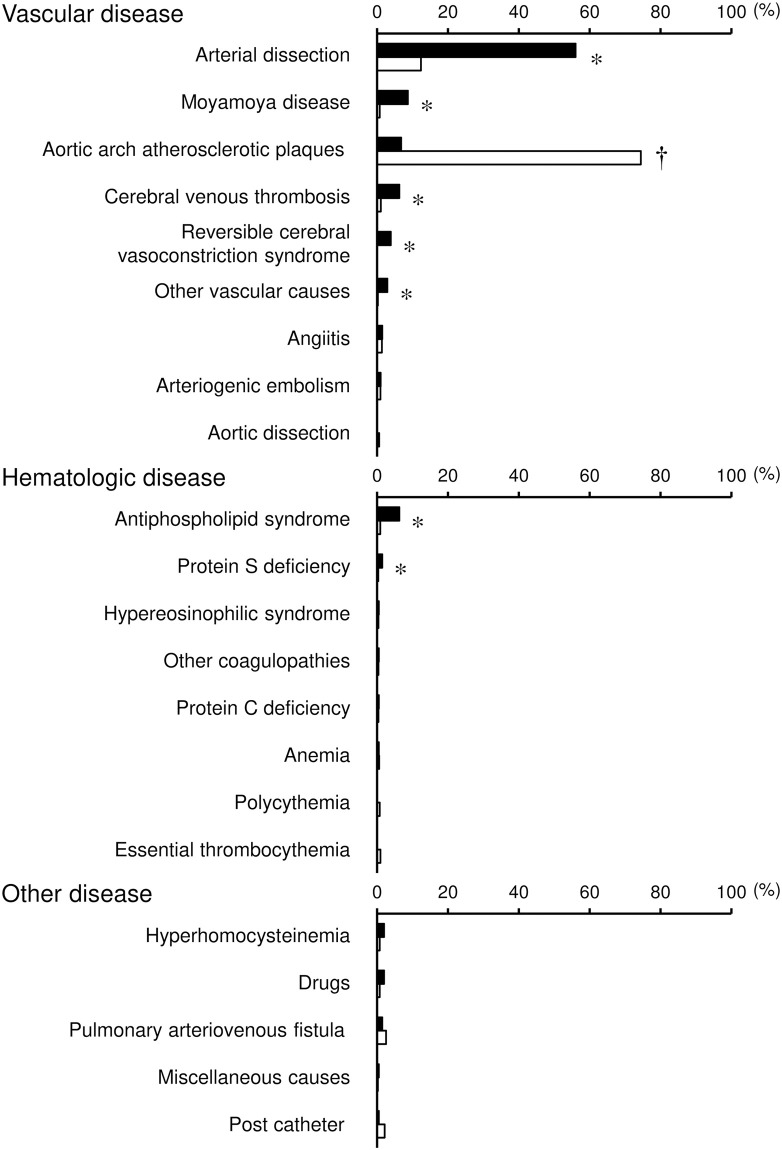
Frequency of uncommon causes in young adults and non-young adults. Frequency of uncommon causes in young adults (closed columns) and non-young adults (open columns) is shown according to origin (vascular disease, hematologic disease, and other disease) among patients with ischemic stroke due to other determined etiologies. When patients had multiple uncommon causes, all potential causes were listed. *P < 0.05, higher in young adults than in non-young adults. †P < 0.05, lower in young adults than in non-young adults.

Based on these findings, several uncommon causes were identified as other determined etiologies of ischemic stroke in the overall patients (Fig 5, S4 Table). Of these, five vascular diseases (arterial dissection, moyamoya disease, cerebral venous thrombosis, reversible cerebral vasoconstriction syndrome, and other vascular causes) were more frequently found in young adults than in non-young adults. Among these vascular diseases, arterial dissection was the most common cause in young adults and showed an age-dependent decrease, even in young adults (Fig 3, S2 Table). Additionally, two hematologic diseases (antiphospholipid syndrome and protein S deficiency) were identified as more frequent causes in young patients than in non-young patients. By contrast, aortic arch atherosclerotic plaques were less frequent in young adults than in non-young adults.

All of the uncommon causes that were more frequent in young adults than in non-young adults showed age-dependent decreasing trends in their frequency (S5 Fig, S4 Table). However, aortic arch atherosclerotic plaques became more frequent with advancing age.

### Sex differences in embolic sources and uncommon causes in young adults

Finally, we investigated sex differences in embolic sources or uncommon causes in young adults (S5 Table). Arterial dissection was more frequent in male young adults than in female young adults, whereas moyamoya disease and antiphospholipid syndrome were more frequent in female young adults than in male young adults.

## Discussion

The major findings of the present study were as follows. (1) Conventional cardiovascular risk factors were less frequent in young adults than in non-young adults, whereas the frequencies of hypertension, diabetes mellitus, and dyslipidemia increased with age, even in young adults. (2) Lifestyle-related risk factors such as smoking, drinking, and obesity were more frequent in young adults than in non-young adults. (3) Cardioembolism and large-artery atherosclerosis were less frequent, whereas other determined etiologies and undetermined etiology were more frequent, in young adults than in non-young adults. Furthermore, thrombotic strokes, including small-vessel occlusion and large-artery atherosclerosis, increased in frequency with advancing age, even in young adults. (4) Some high-risk embolic sources (e.g., arterial myxoma, dilated cardiomyopathy, and intra-cardiac thrombus) and medium-risk embolic sources (e.g., atrial septal defect, nonbacterial thrombotic endocarditis, patent foramen ovale, and left ventricular hypokinesis) were more predominant causes in young adults than in non-young adults, the frequency of which decreased with advancing age. (5) Some uncommon causes (e.g., vascular origin: reversible cerebral vasoconstriction syndrome, moyamoya disease, other vascular origin, arterial dissection, and cerebral venous thrombosis; hematologic diseases: antiphospholipid syndrome and protein S deficiency) were more frequent in young adults than in non-young adults and became less frequent with advancing age. Overall, these findings suggest that embolic sources and uncommon causes are often causes of ischemic stroke in young adults, while conventional risk factors and lifestyle-related factors also contribute to developing ischemic stroke with increasing age, even in young adults.

### Vascular risk factors

We performed a literature review of stroke in young adults (S6 Table) and compared our data with previous studies (S7 Table). Many studies have been performed to elucidate the clinical features of ischemic stroke in young adult patients [4–29]. However, these studies mainly investigated data derived from cohorts of young adult patients alone. As a result, their findings highly varied depending on the study.

The prevalence of vascular risk factors in young adults seems to be higher in our study than that in previous studies (hypertension [4–17, 19–21, 23–29], diabetes mellitus [4–17, 19–21, 23–29], dyslipidemia, and smoking [5–17, 19–21, 23–29], S6 and S7 Tables). Thus, the contribution of vascular risk factors to ischemic stroke may not be negligible in young adults, especially those aged >40 years. Optimal treatment and control of traditional risk factors are top priorities, even in young adults, for preventing development of stroke in young people. In addition to conventional vascular risk factors, lifestyle-related risk factors such as smoking, drinking, and obesity were more frequent in young adults than in non-young adults in our cohorts. Because these modifiable risk factors may contribute to development of ischemic stroke in young adults [35, 36], lifestyle changes to improve health (e.g., smoking cessation, obesity prevention, and moderate drinking) should be recommended in young adults to reduce their stroke risk.

### Stroke subtype

Compared with previous studies [4–29], cardioembolism seemed to be infrequent in our study, whereas small-vessel occlusion was rather common (S6 and S7 Tables). Other studies have also reported a high proportion of small-vessel diseases in Asian patients. In our literature review, the median (interquartile range) proportion of small-vessel occlusion was 19% (14%–24%) in Asian patients [5, 6, 14, 15, 23, 26, 27, 29], but was 11% (8%–15%) in non-Asian patients [4, 7–13, 16–22, 24, 25, 28]. Thus, there may be regional, racial, or ethnic differences in stroke subtypes, especially for small-vessel occlusion. Regarding other subtypes, the proportions of large-artery atherosclerosis [4–29], other determined etiologies [4–29], and undetermined etiologies [4–13, 15–29] were comparable with those reported in previous studies (S6 and S7 Tables).

In the present study, the proportion of cardioembolism and other determined etiologies decreased with age among young adult patients, suggesting that ischemic stroke in younger patients is predominantly caused by embolic sources and uncommon causes. Nevertheless, because the proportions of small-vessel occlusion and large-artery atherosclerosis increased with age even among young adult patients, ischemic stroke may be partly attributable to vascular damage due to vascular risk factors even in young adults with advancing age.

### Embolic sources of cardioembolism

In the present study, the implementation rate of diagnostic tests differed significantly between young adults and non-young adults. Although this difference may be unavoidable during the diagnostic process in daily clinical practice, it may have led to apparent differences in the frequencies of potential causes. Because cardiac embolic sources are often unidentified by routine examination, transesophageal echocardiography and prolonged electrocardiogram would be required to identify potential causes when stroke is considered to be caused by an embolic mechanism.

The present study supports the idea that dilated cardiomyopathy [10–13, 15, 17, 18, 23, 27], atrial myxoma [4, 7, 9, 12, 18, 27], patent foramen ovale [4–10, 12, 13, 15–18, 20, 23, 28], and atrial septal defect [4, 8, 9] are actually more frequent causes of cardioembolism in young adults than in non-young adults despite their low frequency. The etiological importance of patent foramen ovale must be interpreted with caution [37] because its high frequency in young adults potentially arises from the higher prevalence [38] or detection bias due to the higher implementation rate of transesophageal echocardiography in young adults than in non-young adults [39]. Moreover, additional data on the anatomical and functional characteristics of high-risk patent foramen ovale are required to determine whether a patent foramen ovale was actually the most likely cause of ischemic stroke in individual patients [40]. In our cohort, nonbacterial thrombotic endocarditis was additionally identified as a more frequent cause in young adults than in non-young adults, which may draw attention to possible cardioembolism in young adults with comorbidities potentially causing a hypercoagulable state. Although atrial fibrillation was far less frequent in young adults than in non-young adults, it was the most common embolic source of cardioembolism even among young adults [4, 6–9, 11–17, 19–21, 23, 26–29]. The clinical significance of atrial fibrillation cannot be disregarded even in young adults, especially those aged >30 years.

### Uncommon causes as other determined etiologies

In the present study, undetermined etiologies were found more frequently in young adults than in non-young adults, indicating that further studies are required in the young age group. Among the uncommon causes, the frequency of arterial dissection was particularly high in our study, suggesting that dissection is the most common “rare” cause of ischemic stroke in young adults. The frequency was within the range of that reported in previous studies (S6 and S7 Tables) [4–13, 15–19, 21–23, 25, 27–29]; however, the reported frequency highly varies among previous studies, possibly because of the different implementation rates of MR imaging and MR angiography [41]. Careful investigation of the vascular wall or its shape is necessary using CT angiography, MR angiography, and/or conventional angiography when young adults develop ischemic stroke of unknown cause. Moyamoya disease was the second most common cause of ischemic stroke in young adults [4–6, 10–13, 15, 16, 18, 19, 23, 27–29], which may reflect the higher prevalence of moyamoya disease in Asia [42, 43]. Cerebral venous thrombosis was also identified as a more frequent cause in young adults than in non-young adults [4, 10, 13, 27]. An increased risk of thrombosis in cerebral veins should be taken into account if young adults have a hypercoagulable state, such as that induced by pregnancy, puerperium, oral contraceptives, and thrombophilia [44]. Reversible cerebral vasoconstriction syndrome [4–6, 8–12, 15–19, 27] was detected mostly in young adults. However, the potential involvement of vasoconstriction in causing ischemic stroke may have been underestimated in both age groups because the associated causes (e.g., migraine and prescription medications) were not necessarily investigated in all patients. Nevertheless, its frequency was far higher in young adults than in non-young adults. Its potential existence requires exclusion especially in middle-aged women, after vasoactive drugs, or postpartum [34].

In the present study, hematologic studies were presumably performed when abnormal findings were detected in a blood coagulation test or when potential causes were unidentified even after extensive investigation. Moreover, we retrospectively collected data on hematologic diseases (e.g., antiphospholipid syndrome) after they were definitively diagnosed after discharge. These biases may have affected the prevalence of hematologic diseases in our patients. Under such conditions, antiphospholipid syndrome [4–7, 9–12, 15–20, 23, 25, 27, 29] and protein S deficiency [9, 10, 12, 15, 17, 20] were more frequent in young adults than in non-young adults, whereas the frequencies of hyperhomocysteinemia [4, 11, 15–17, 20, 26, 27, 29], drugs [11, 12, 15, 27], and protein C deficiency [8, 9, 12, 20] did not differ between young adults and non-young adults. These findings suggest that uncommon causes predominantly originated from vascular disorders in young adults with ischemic stroke but that hematological causes should be explored after ruling out the existence of vascular diseases.

In the present cohort, hereditary diseases such as cerebral autosomal dominant arteriopathy with subcortical infarcts and leukoencephalopathy and Fabry disease were not detected during the study period. Cerebral autosomal dominant arteriopathy with subcortical infarcts and leukoencephalopathy should be suspected when patients have a family history and migraine with aura [45]. Complications of heart failure and renal failure, as well as ectasia and elongation of the basilar artery, are helpful for diagnosing Fabry disease [46]. A genetic approach is also required to further clarify the etiology of stroke in young adults.

Sex differences are of interest for stroke in young adults because hormonal differences can be underlying uncommon causes. In the present study, the frequency of arterial dissection was higher, while the frequency of moyamoya disease and antiphospholipid syndrome were lower, in male young adults than in female young adults. While some studies have reported no sex differences in arterial dissection [47, 48], an epidemiological study in Japan found that arterial dissection was a more common cause in male young adults than in female young adults [49]. Moyamoya disease [42] and antiphospholipid antibody syndrome [50] were also suggested to be more prevalent in young women than in young men, which may contribute to sex differences in stroke in young adults. Nevertheless, the small sample size in the present study precludes us from confirming these differences. Thus, accumulation of more young adult patients with stroke is required to validate our findings.

### Study limitations

The present study had several limitations that should be considered. First, not all diagnostic assessments were performed in all patients; therefore, potential embolic sources and rare causes could have been unrecognized. Additionally, screening tests are insufficient, especially for hematologic diseases and genetic diseases. Consequently, the differences detected in this study may have been affected by the differences in the rate of required assessments. Second, the clinical features of ischemic stroke were retrospectively verified, leading to misclassification; by contrast, the diagnostic criteria were standardized, and the causes were prespecified before starting our registry study. Third, the participants in this study were enrolled for >10 years, during which the detection rate of various etiologies may have changed with advancements in diagnostic techniques. Finally, this study included only Japanese patients hospitalized in a restricted geographic region, which limits the generalizability of our findings.

## Conclusions

The present study verified disproportional frequencies of certain embolic sources and uncommon causes according to age as potential causes of ischemic stroke. These findings will be helpful for clinicians to prevent stroke in young adults or to identify causes of ischemic stroke in young adults. Further studies are warranted to validate age-specific changes in the causes of ischemic stroke in different settings, races, and ethnic groups.

## Supporting information

S1 Fig Flow chart of patient selection. (PDF)

S2 Fig Diagnostic process for stroke subtype. (PDF)

S3 Fig Distribution of patients according to age. Frequencies of patients are shown in 5-year age groups (closed columns: young adults, open columns: non-young adults).
(PDF)

S4 Fig Frequency of embolic sources of cardioembolism in 10-year age groups. Frequencies of embolic sources that were more frequent in young adults than in non-young adults and had significant trends with age are shown by percentages among patients with ischemic stroke due to cardioembolism according to 10-year age groups (closed columns: young adults, open columns: non-young adults). All embolic sources are listed when multiple embolic sources were detected in one patient. P values indicate P values for trends according to 10-year age groups in overall patients.
(PDF)

S5 Fig Frequency of uncommon causes as other determined etiologies in 10-year age groups. Frequencies of uncommon causes that were more frequent in young adults than in non-young adults and had significant trends with age are shown by percentages among patients with ischemic stroke due to other determined etiologies according to 10-year age groups (closed columns: young adults, open columns: non-young adults). All uncommon causes are listed when multiple uncommon causes were detected in one patient. P values indicate P values for trends according to 10-year age groups in overall patients.
(PDF)

S1 Table Prevalence of vascular risk factors according to age among young adults. (PDF)

S2 Table Proportions of stroke subtypes according to age among young adults. (PDF)

S3 Table Embolic sources of ischemic stroke due to cardioembolism in young adults and non-young adults. (PDF)

S4 Table Uncommon causes of ischemic stroke due to other determined etiologies in young adults and non-young adults. (PDF)

S5 Table Sex differences in embolic sources or uncommon causes in young adults. (PDF)

S6 Table Literature review of stroke in young adults. (PDF)

S7 Table Summary of a literature review for clinical features of stroke in young adults. (PDF)

S1 Appendix Fukuoka stroke registry investigators. (DOCX)
