# Supplementary figures and images for: Causes of ischemic stroke in young adults versus non-young adults: A multicenter hospital-based observational study
Source: PLoS One. 2022 Jul 13;17(7):e0268481. doi: 10.1371/journal.pone.0268481 (PMC9278748; doi:10.1371/journal.pone.0268481)

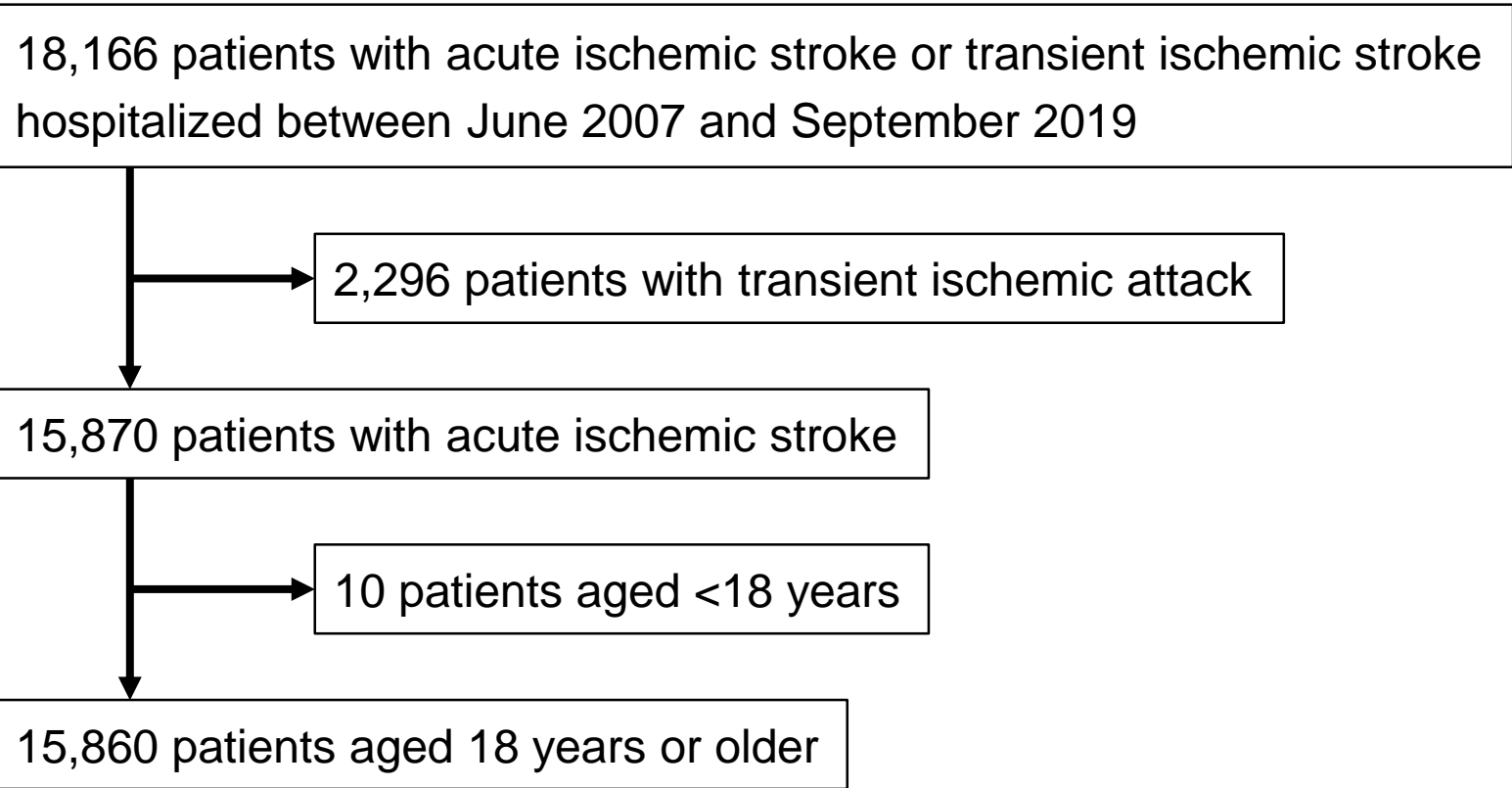

Supplement: S1 Fig — (PDF) [file pone.0268481.s001.pdf]

Acute ischemic stroke

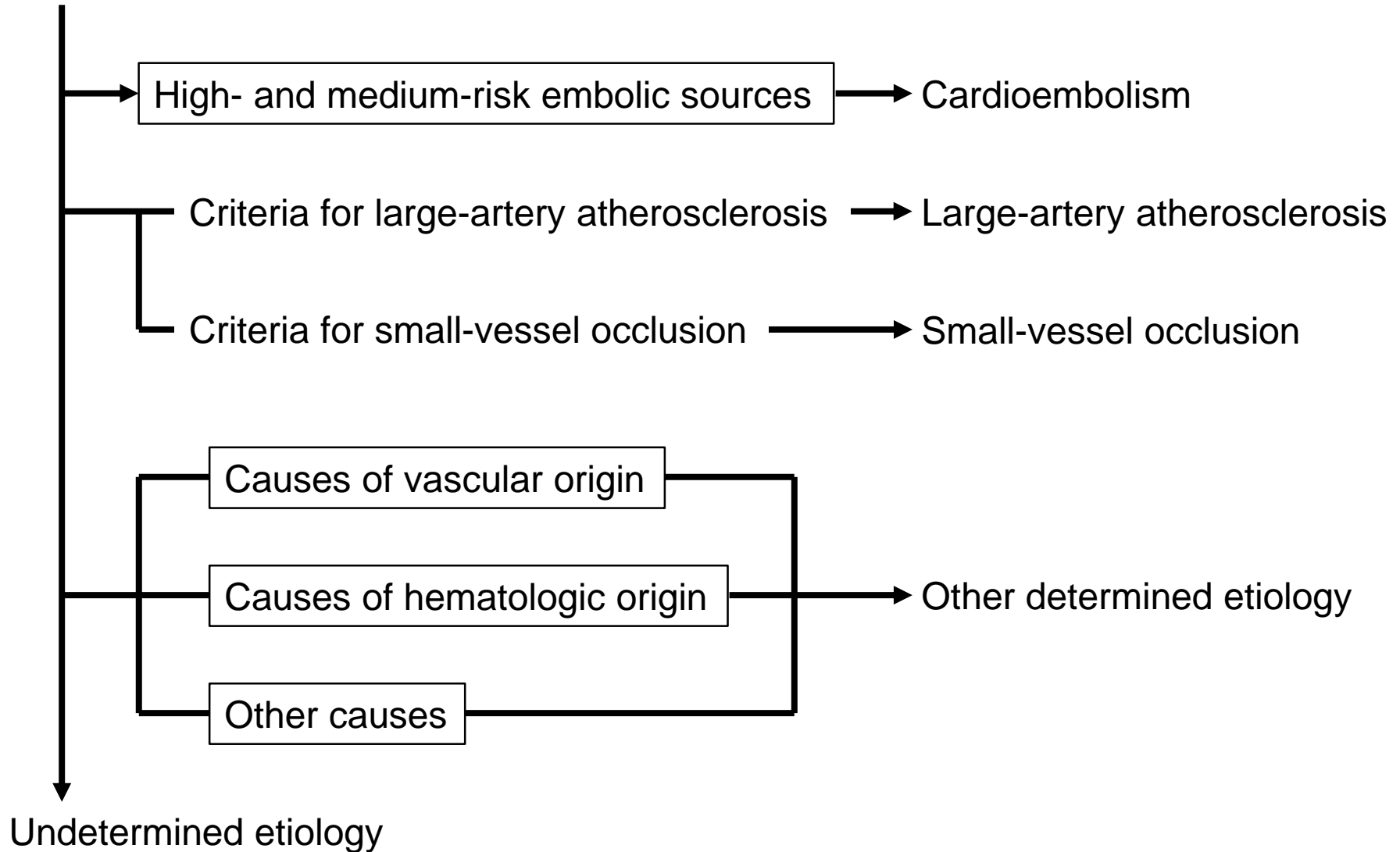

Supplement: S2 Fig — (PDF) [file pone.0268481.s002.pdf]

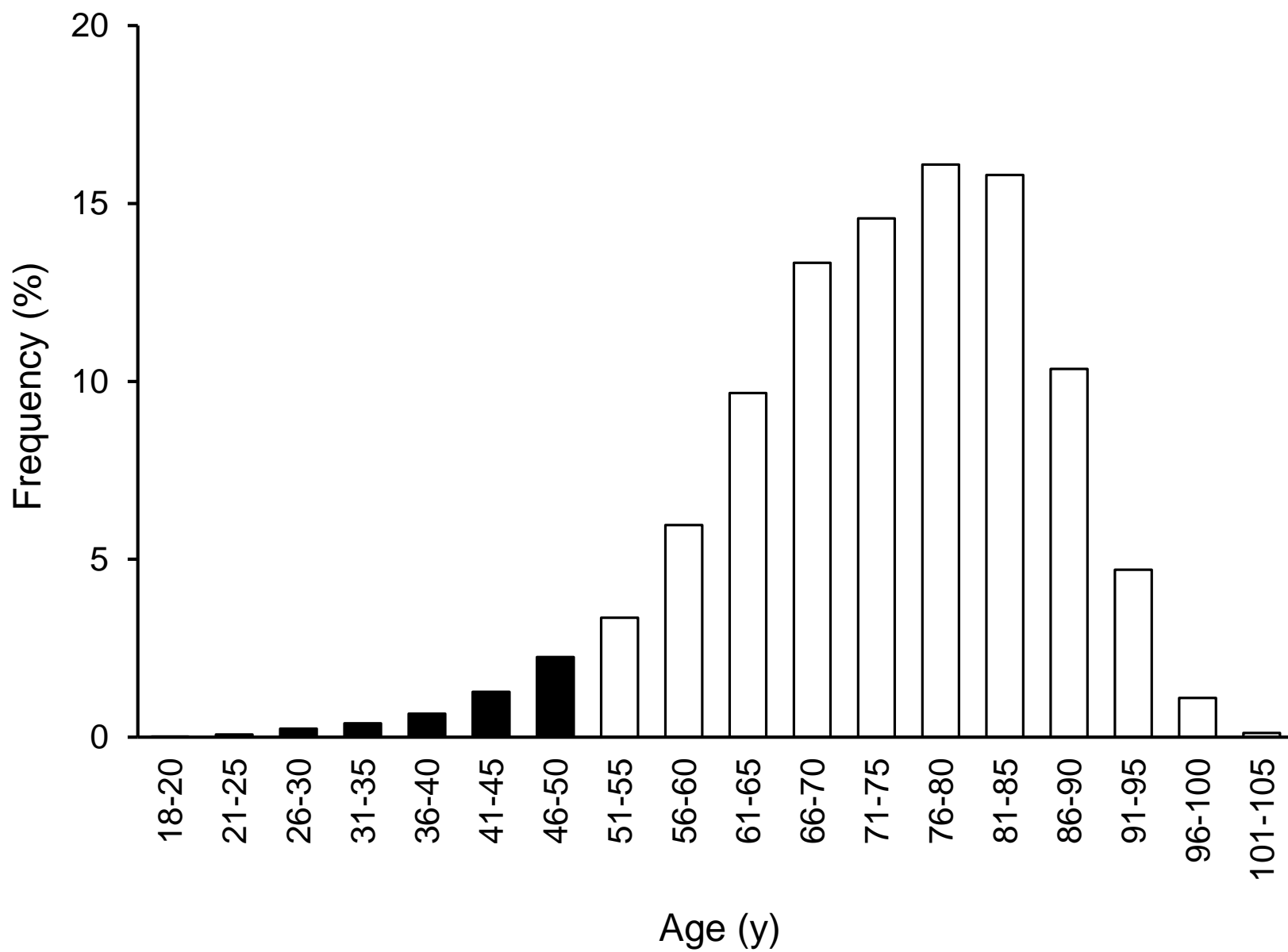

Supplement: S3 Fig — Frequencies of patients are shown in 5-year age groups (closed columns: young adults, open columns: non-young adults). (PDF) [file pone.0268481.s003.pdf]
