# Supplementary material for: Causes of ischemic stroke in young adults versus non-young adults: A multicenter hospital-based observational study
Source: PLoS One. 2022 Jul 13;17(7):e0268481. doi: 10.1371/journal.pone.0268481 (PMC9278748; doi:10.1371/journal.pone.0268481)
Supplement: S4 Fig — Frequencies of embolic sources that were more frequent in young adults than in non-young adults and had significant trends with age are shown by percentages among patients with ischemic stroke due to cardioembolism according to 10-year age groups (closed columns: young adults, open columns: non-young adults). All embolic sources are listed when multiple embolic sources were detected in one patient. P values indicate P values for trends according to 10-year age groups in overall patients. (PDF) [file pone.0268481.s004.pdf]

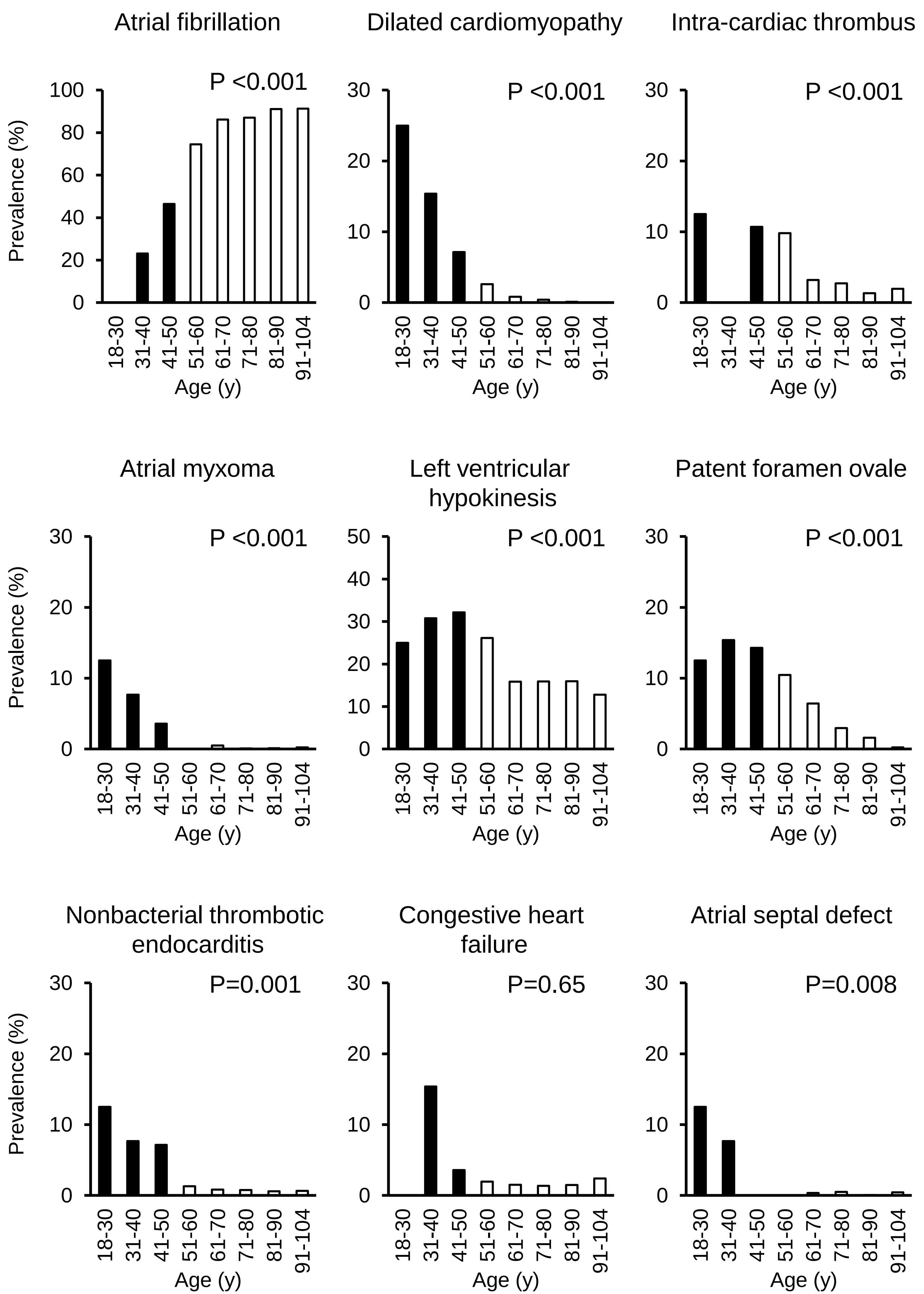

Atrial fibrillation
Dilated cardiomyopathy
Intra-cardiac thrombus
Atrial myxoma
Left ventricular hypokinesis
Patent foramen ovale
Nonbacterial thrombotic endocarditis
Congestive heart failure
Atrial septal defect
P <0.001
P=0.001
P=0.65
P=0.008
Prevalence (%)
Age (y)
18-30
31-40
41-50
51-60
61-70
71-80
81-90
91-104
