# Supplementary material for: Causes of ischemic stroke in young adults versus non-young adults: A multicenter hospital-based observational study
Source: PLoS One. 2022 Jul 13;17(7):e0268481. doi: 10.1371/journal.pone.0268481 (PMC9278748; doi:10.1371/journal.pone.0268481)
Supplement: S5 Fig — Frequencies of uncommon causes that were more frequent in young adults than in non-young adults and had significant trends with age are shown by percentages among patients with ischemic stroke due to other determined etiologies according to 10-year age groups (closed columns: young adults, open columns: non-young adults). All uncommon causes are listed when multiple uncommon causes were detected in one patient. P values indicate P values for trends according to 10-year age groups in overall patients. (PDF) [file pone.0268481.s005.pdf]

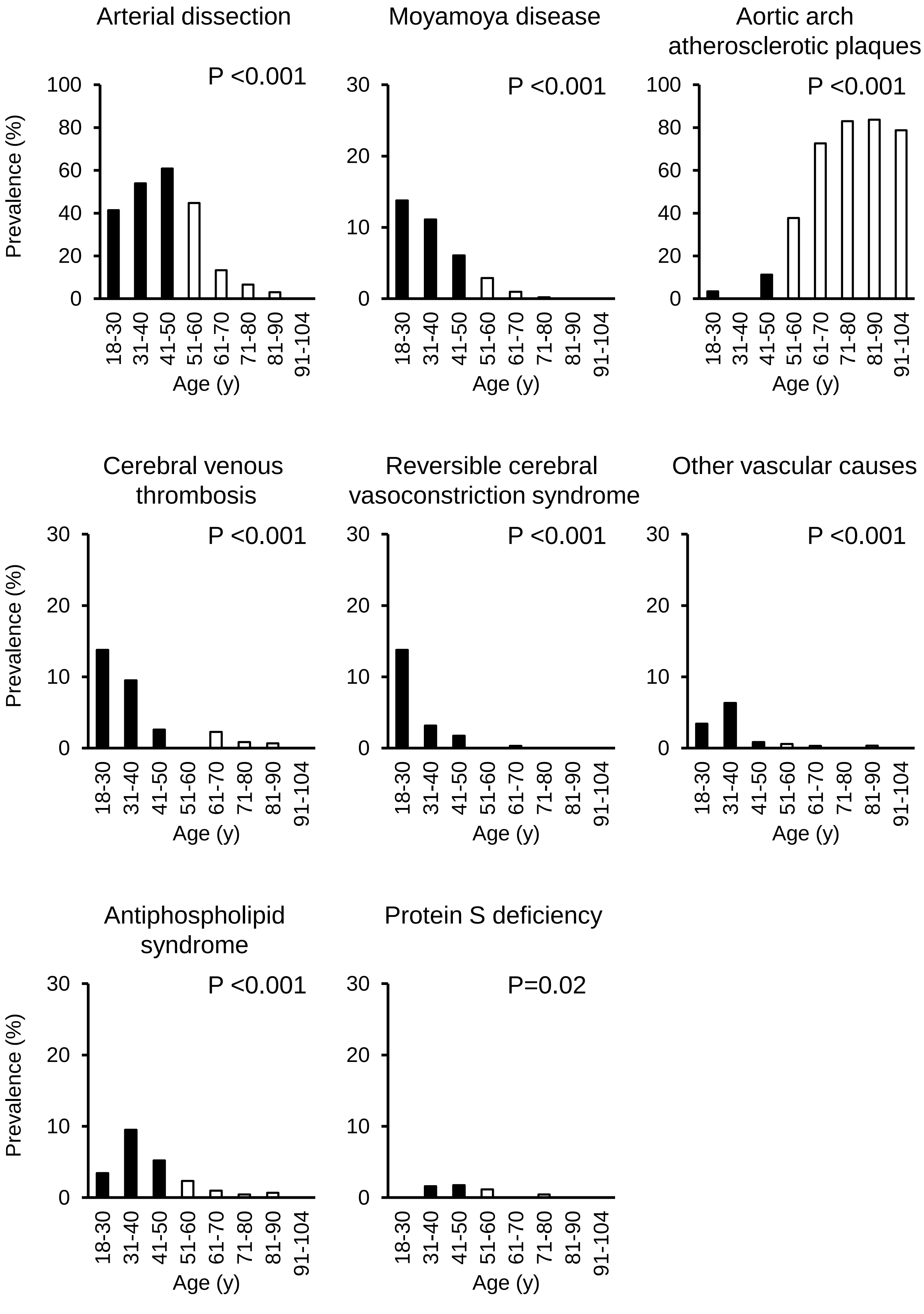

Arterial dissection
P <0.001
Moyamoya disease
P <0.001
Aortic arch atherosclerotic plaques
P <0.001
Cerebral venous thrombosis
P <0.001
Reversible cerebral vasoconstriction syndrome
P <0.001
Other vascular causes
P <0.001
Antiphospholipid syndrome
P <0.001
Protein S deficiency
P=0.02
Prevalence (%)
Age (y)
18-30
31-40
41-50
51-60
61-70
71-80
81-90
91-104
