# Supplementary material for: Causes of ischemic stroke in young adults versus non-young adults: A multicenter hospital-based observational study
Source: PLoS One. 2022 Jul 13;17(7):e0268481. doi: 10.1371/journal.pone.0268481 (PMC9278748; doi:10.1371/journal.pone.0268481)
Supplement: S1 Table — (PDF) [file pone.0268481.s006.pdf]

S1 Table. Prevalence of vascular risk factors according to age among young adults.

	18–30 y	31–35 y	36–40 y	41–45 y	46–50 y	P_{trend}
Hypertension	6 (11.3)*	20 (32.3)*	57 (54.3)*	123 (60.9)*	246 (68.9)*	<0.001
Diabetes mellitus	5 (9.4)*	6 (9.7)*	19 (18.1)*	49 (24.3)	105 (29.4)	<0.001
Dyslipidemia	7 (13.2)*	14 (22.6)*	45 (42.9)	95 (47.0)	212 (59.4)	<0.001
Smoking	21 (39.6)	38 (61.3)	69 (65.7)*	129 (63.9)*	243 (68.1)*	<0.001
Drinking	15 (28.3)	15 (24.2)	40 (38.1)	84 (41.6)*	173 (48.5)*	<0.001
Obesity	14 (26.4)	24 (38.7)	48 (45.7)*	102 (51.5)*	155 (43.5)*	0.07

P_{trend}: P for trend.

Data are expressed as n (%). P_{trend} was tested for the proportion of risk factors according to 5-year age increments among young adults. *$P < 0.05$ vs. non-young adults by multiple comparison.
