# Supplementary material for: Causes of ischemic stroke in young adults versus non-young adults: A multicenter hospital-based observational study
Source: PLoS One. 2022 Jul 13;17(7):e0268481. doi: 10.1371/journal.pone.0268481 (PMC9278748; doi:10.1371/journal.pone.0268481)
Supplement: S2 Table — (PDF) [file pone.0268481.s007.pdf]

S2 Table. Proportions of stroke subtypes according to age among young adults.

	18–30 y	31–35 y	36–40 y	41–45 y	46–50 y	P_{trend}
Cardioembolism	8 (15.1)	4 (6.5)	9 (8.6)	8 (4.0)	20 (5.6)	0.02
High-risk sources	5 (9.4)	1 (1.6)	4 (3.8)	6 (3.0)	15 (4.2)	0.48
Medium-risk sources	3 (5.7)	3 (4.8)	5 (4.8)	2 (1.0)	5 (1.4)	0.01
Large-artery atherosclerosis	0 (0.0)	2 (3.2)	8 (7.6)	23 (11.4)	49 (13.7)	<0.001
Small-vessel occlusion	2 (3.8)	11 (17.7)	28 (26.7)	61 (30.2)	138 (38.7)	<0.001
Other determined etiology	29 (54.7)	28 (45.2)	35 (33.3)	51 (25.3)	64 (17.9)	<0.001
Arterial dissection	12 (22.6)	14 (22.6)	20 (19.1)	32 (15.8)	38 (10.6)	<0.001
Undetermined cause	14 (26.4)	17 (27.4)	25 (23.8)	59 (29.2)	86 (24.1)	0.64

P_{trend}: P for trend.

Data are expressed as n (%). P_{trend} was tested for the proportions of stroke subtypes according to 5-year age increments among young adults.
