# Supplementary material for: Causes of ischemic stroke in young adults versus non-young adults: A multicenter hospital-based observational study
Source: PLoS One. 2022 Jul 13;17(7):e0268481. doi: 10.1371/journal.pone.0268481 (PMC9278748; doi:10.1371/journal.pone.0268481)
Supplement: S3 Table — (PDF) [file pone.0268481.s008.pdf]

S3 Table. Embolic sources of ischemic stroke due to cardioembolism in young adults and non-young adults.

	Young adults, n = 49	Non-young adults, n = 3883	OR (95% CI)	P	P_{trend}
High-risk embolic sources					
Atrial fibrillation	16 (32.7)	3435 (88.5)	0.06 (0.03–0.12)	<0.001	<0.001
Atrial fibrillation without mitral stenosis	16 (32.7)	3384 (87.2)	0.07 (0.04–0.13)	<0.001	<0.001
Atrial fibrillation with mitral stenosis	0 (0.0)	51 (1.3)	–	1.00	0.95
Dilated cardiomyopathy	6 (12.2)	16 (0.4)	33.72 (12.59–90.33)	<0.001	<0.001
Intracardiac thrombus	4 (8.2)	95 (2.5)	3.54 (1.25–10.05)	0.02	<0.001
Atrial myxoma	3 (6.1)	7 (0.2)	36.11 (9.05–144.03)	<0.001	<0.001
Left ventricular akinesis	3 (6.1)	133 (3.4)	1.84 (0.56–5.99)	0.31	0.006
Sick sinus syndrome	3 (6.1)	211 (5.4)	1.13 (0.35–3.68)	0.83	<0.001
Mechanical prosthetic valve	2 (4.1)	44 (1.1)	3.71 (0.87–15.76)	0.08	<0.001
Infective endocarditis	1 (2.0)	19 (0.5)	4.24 (0.56–32.29)	0.16	0.04
Recent myocardial infarction	0 (0.0)	13 (0.3)	–	1.00	0.03
Medium-risk embolic sources					
Left ventricular hypokinesis	15 (30.6)	619 (15.9)	2.33 (1.26–4.30)	0.007	<0.001
Patent foramen ovale	7 (14.3)	114 (2.9)	5.51 (2.42–12.53)	<0.001	<0.001
Nonbacterial thrombotic endocarditis	4 (8.2)	29 (0.8)	11.81 (3.99–34.99)	<0.001	0.001
Congestive heart failure	3 (6.1)	61 (1.6)	4.09 (1.24–13.50)	0.02	0.65
Left atrial turbulence	3 (6.1)	262 (6.8)	0.90 (0.28–2.92)	0.86	<0.001
Atrial septal defect	2 (4.1)	11 (0.3)	14.98 (3.23–69.44)	0.001	0.008
Bioprosthetic cardiac valve	1 (2.0)	34 (0.9)	2.36 (0.32–17.58)	0.40	0.88
Atrial septal aneurysm	1 (2.0)	35 (0.9)	2.29 (0.31–17.06)	0.42	0.002
Old myocardial infarction	0 (0.0)	1 (0.0)	–	1.00	0.55
Mitral stenosis without atrial fibrillation	0 (0.0)	8 (0.2)	–	1.00	0.93
Mitral valve prolapse	0 (0.0)	43 (1.1)	–	1.00	0.20
Mitral annulus calcification	0 (0.0)	43 (1.1)	–	1.00	<0.001
Atrial flutter	0 (0.0)	73 (1.9)	–	1.00	0.16

OR: odds ratio, CI: confidence interval, P_{trend}: P for trend.

Data are presented as n (%). OR and 95% CI of each embolic source in young adults were estimated in reference to non-young adults. When patients had multiple embolic sources, all potential sources were listed. P_{trend} was tested according to 10-year age increments in all patients.
