# Supplementary material for: Causes of ischemic stroke in young adults versus non-young adults: A multicenter hospital-based observational study
Source: PLoS One. 2022 Jul 13;17(7):e0268481. doi: 10.1371/journal.pone.0268481 (PMC9278748; doi:10.1371/journal.pone.0268481)
Supplement: S4 Table — (PDF) [file pone.0268481.s009.pdf]

S4 Table. Uncommon causes of ischemic stroke due to other determined etiologies in young adults and non-young adults.

	Young adults, n = 207	Non-young adults, n = 1278	OR (95% CI)	P	P_{trend}
Vascular origin					
Arterial dissection	116 (56.0)	158 (12.4)	9.04 (6.55–12.46)	<0.001	<0.001
Moyamoya disease	18 (8.7)	9 (0.7)	13.43 (5.95–30.33)	<0.001	<0.001
Aortic arch atherosclerotic plaques	14 (6.8)	951 (74.4)	0.02 (0.01–0.04)	<0.001	<0.001
Cerebral venous thrombosis	13 (6.3)	13 (1.0)	6.52 (2.98–14.27)	<0.001	<0.001
Reversible cerebral vasoconstriction syndrome	8 (3.9)	1 (0.1)	51.34 (6.39–412.66)	<0.001	<0.001
Other vascular causes	6 (2.9)	3 (0.2)	12.69 (3.15–51.13)	<0.001	<0.001
Angiitis	3 (1.5)	17 (1.3)	1.09 (0.32–3.76)	0.89	0.87
Arteriogenic embolism	2 (1.0)	12 (0.9)	1.03 (0.23–4.63)	0.97	0.90
Aortic dissection	0 (0.0)	7 (0.6)	–	0.60	0.48
Hematologic origin					
Antiphospholipid syndrome	13 (6.3)	11 (0.9)	7.72 (3.41–17.47)	<0.001	<0.001
Protein S deficiency	3 (1.5)	4 (0.3)	4.68 (1.04–21.08)	0.04	0.02
Hypereosinophilic syndrome	1 (0.5)	5 (0.4)	1.24 (0.14–10.63)	0.85	0.96
Other coagulopathies	1 (0.5)	5 (0.4)	1.24 (0.14–10.63)	0.85	0.56
Protein C deficiency	1 (0.5)	5 (0.4)	1.24 (0.14–10.63)	0.85	0.20
Anemia	1 (0.5)	7 (0.6)	0.88 (0.11–7.20)	0.91	0.92
Polycythemia	0 (0.0)	9 (0.7)	–	0.62	0.70
Essential thrombocythemia	0 (0.0)	12 (0.9)	–	0.39	0.03
Other causes					
Hyperhomocysteinemia	4 (1.9)	9 (0.7)	2.78 (0.85–9.11)	0.09	0.006
Drugs	4 (1.9)	9 (0.7)	2.78 (0.85–9.11)	0.09	0.72
Pulmonary arteriovenous fistula	3 (1.5)	32 (2.5)	0.57 (0.17–1.89)	0.36	0.003
Miscellaneous causes	1 (0.5)	3 (0.2)	2.06 (0.21–19.93)	0.53	0.27
Post-catheter	1 (0.5)	27 (2.1)	0.22 (0.03–1.66)	0.14	0.005

OR: odds ratio, CI: confidence interval, P_{trend}: P for trend.

Data are presented as n (%). OR and 95% CI of each uncommon cause in young adults were estimated in reference to non-young adults. When patients had multiple uncommon causes, all potential causes were listed. P_{trend} was tested according to 10-year age increments in all patients.
