# Supplementary material for: Causes of ischemic stroke in young adults versus non-young adults: A multicenter hospital-based observational study
Source: PLoS One. 2022 Jul 13;17(7):e0268481. doi: 10.1371/journal.pone.0268481 (PMC9278748; doi:10.1371/journal.pone.0268481)
Supplement: S5 Table — (PDF) [file pone.0268481.s010.pdf]

S5 Table. Sex differences in embolic sources or uncommon causes in young adults.

	Men	Women	P
Embolic sources	n = 49	n = 13	
Atrial fibrillation	14 (38.9)	2 (15.4)	0.17
Left ventricular hypokinesis	13 (36.1)	2 (15.4)	0.29
Uncommon causes	n = 120	n = 87	
Arterial dissection	83 (69.2)	33 (37.9)	<0.001
Moyamoya disease	6 (5.0)	12 (13.8)	0.03
Aortic arch atherosclerotic plaques	11 (9.2)	3 (3.5)	0.11
Cerebral venous thrombosis	5 (4.2)	8 (9.2)	0.14
Antiphospholipid syndrome	4 (3.3)	9 (10.3)	0.04

Data are presented as n (%). When patients with cardioembolism or other determined etiologies had multiple causes, all potential causes were listed. The frequencies of embolic sources or uncommon causes were compared between sexes in young adult patients. A comparison was performed for embolic sources and uncommon causes found in more than 10 young adult patients.
