# Supplementary material for: Causes of ischemic stroke in young adults versus non-young adults: A multicenter hospital-based observational study
Source: PLoS One. 2022 Jul 13;17(7):e0268481. doi: 10.1371/journal.pone.0268481 (PMC9278748; doi:10.1371/journal.pone.0268481)
Supplement: S6 Table — (PDF) [file pone.0268481.s011.pdf]

S6 Table. Literature review of stroke in young adults.

	Country	Study period	N	Age, y
Varona et al. [10]	Spain	1974–2002	272	15–45
Rutten-Jacobs et al. [16]	Netherlands	1980–2010	447	18–50
Yesilot Barlas et al. [18]	Europe	1988–2010	3331	15–49
Jovanovic et al. [11]	Serbia	1989–2005	865	15–45
Rasura et al. [9]	Italy	1992–2001	394	14–47
Chan et al. [4]	Canada	1993–1997	356	15–45
Kwon et al. [5]	Korea	1994–1997	149	15–44
Cerrato et al. [7]	Italy	1994–2001	273	16–49
Putaala et al. [12]	Finland	1994–2007	1008	15–49
Lee et al. [6]	Taiwan	1997–2001	241	18–45
Nedeltchev et al. [8]	Switzerland	1997–2002	203	16–45
Spengos et al. [13]	Greece	1999–2008	253	14–45
Tancredi et al. [17]	Italy	2000–2005	324	16–44
Zhang et al. [14]	China	2001–2010	669	18–45
Ji et al. [15]	China	2005–2010	215	18–45
Renna et al. [20]	Italy	2005–2013	150	<50
Jaffre et al. [19]	France	2006–2012	400	16–54
Nakagawa et al. [24]	United States	2006–2012	427	≤55
Tang et al. [29]	China	2007–2018	411	18–50
Goeggel Simonetti et al. [21]	Switzerland	2008–2012	624	16–55
Li et al. [23]	China	2008–2014	1395	18–45
Fromm et al. [22]	Norway	2010–2012	150	15–60
Shaban et al. [28]	United States	2010–2016	322	15–45
Huang et al. [26]	China	2012–2015	961	15–45
Crespo Pimentel et al. [25]	Portugal, Austria	2014–2016	265	18–55
Kono et al. [27]	Japan	2015–2018	519	16–55
Our study	Japan	2007–2019	779	18–50
