# Supplementary material for: Causes of ischemic stroke in young adults versus non-young adults: A multicenter hospital-based observational study
Source: PLoS One. 2022 Jul 13;17(7):e0268481. doi: 10.1371/journal.pone.0268481 (PMC9278748; doi:10.1371/journal.pone.0268481)
Supplement: S7 Table — (PDF) [file pone.0268481.s012.pdf]

S7 Table. Summary of a literature review for clinical features of stroke in young adults.

	Min-Max	Median (IQR)	Ours
Risk factors			
Hypertension	19–64	37 (25–43)	58
Diabetes mellitus	2–33	9 (8–15)	24
Dyslipidemia	8–60	37 (17–47)	48
Atrial fibrillation	0.5–29	3 (1–4)	3
Smoking	29–59	47 (41–50)	64
Stroke subtype			
Cardioembolism	6–47	19 (14–24)	6
Large-artery atherosclerosis	2–43	12 (8–17)	11
Likely large-artery atherosclerosis			
Small-vessel occlusion	3–42	13 (8–18)	31
Other determined cause	6–35	24 (17–28)	26
Undetermined cause	10–50	29 (24–36)	27
Embolic sources			
Patent foramen ovale	3–45	12 (4–17)	8
Dilated cardiomyopathy	0.3–5	2 (1–3)	1
Atrial septal defect	0.3–1	1 (1–1)	1
Atrial myxoma	0.1–1	0.4 (0.2–1)	0.4
Uncommon causes			
Reversible cerebral vasoconstriction syndrome	0.2–7	1 (1–2)	1
Moyamoya disease	0.2–7	2 (1–3)	2
Antiphospholipid antibody syndrome	0.4–13	2 (1–3)	2
Cerebral venous thrombosis	0.4–4	3 (1–4)	2
Arterial dissection	1–25	12 (7–14)	15
Protein S deficiency	0.1–3	0.4 (0.3–1)	1
Hyperhomocysteinemia	0.2–35	4 (0.4–16)	1
Drugs	0.2–2	0.3 (0.3–1)	1
Protein C deficiency	0.1–1	0.4 (0.2–1)	0.3

Min: minimum, Max: maximum, IQR: interquartile range.
Data are expressed as percentage in all young adult patients with ischemic stroke. The percentages reported in previous studies are shown for comparison with those in the present study.
