# Supplementary material for: Causes of ischemic stroke in young adults versus non-young adults: A multicenter hospital-based observational study
Source: PLoS One. 2022 Jul 13;17(7):e0268481. doi: 10.1371/journal.pone.0268481 (PMC9278748; doi:10.1371/journal.pone.0268481)
Supplement: S1 Appendix — (DOCX) [file pone.0268481.s013.docx]

**S1 Appendix. Fukuoka Stroke Registry Investigators**

***Steering Committee and Research Working Group Members:***

Takao Ishitsuka, MD, PhD (Fukuoka Mirai Hospital, Fukuoka, Japan), Setsuro Ibayashi, MD, PhD (Chair, Seiai Rehabilitation Hospital, Onojo, Japan), Kenji Kusuda, MD, PhD (Seiai Rehabilitation Hospital, Onojo, Japan), Kenichiro Fujii, MD, PhD (Japan Seafarers Relief Association Moji Ekisaikai Hospital, Kitakyushu, Japan), Tetsuhiko Nagao, MD, PhD (Safety Monitoring Committee, Seiai Rehabilitation Hospital, Onojo, Japan), Yasushi Okada, MD, PhD (Vice-Chair, National Hospital Organization Kyushu Medical Center, Fukuoka, Japan), Masahiro Yasaka, MD, PhD (National Hospital Organization Kyushu Medical Center, Fukuoka, Japan), Hiroaki Ooboshi, MD, PhD (Fukuoka Dental College Medical and Dental Hospital, Fukuoka, Japan), Takanari Kitazono, MD, PhD (Principal Investigator, Kyushu University, Fukuoka, Japan), Katsumi Irie, MD, PhD (Hakujyuji Hospital, Fukuoka, Japan), Tsuyoshi Omae, MD, PhD (Imazu Red Cross Hospital, Fukuoka, Japan), Kazunori Toyoda, MD, PhD (National Cerebral and Cardiovascular Center, Suita, Japan), Hiroshi Nakane, MD, PhD (National Hospital Organization Fukuoka–Higashi Medical Center, Koga, Japan), Masahiro Kamouchi, MD, PhD (Kyushu University, Fukuoka, Japan), Hiroshi Sugimori, MD, PhD (National Hospital Organization Kyushu Medical Center, Fukuoka, Japan), Shuji Arakawa, MD, PhD (Steel Memorial Yawata Hospital, Kitakyushu, Japan), Kenji Fukuda, MD, PhD (St. Mary’s Hospital, Kurume, Japan), Tetsuro Ago, MD, PhD (Kyushu University, Fukuoka, Japan), Jiro Kitayama, MD, PhD (Fukuoka Red Cross Hospital, Fukuoka, Japan), Shigeru Fujimoto, MD, PhD (Jichi Medical University, Shimotsuke, Japan), Shoji Arihiro, MD (Japan Labor Health and Welfare Organization Kyushu Rosai Hospital, Kitakyushu, Japan), Junya Kuroda, MD, PhD (National Hospital Organization Fukuoka–Higashi Medical Center, Koga, Japan), Yoshinobu Wakisaka, MD, PhD (Kyushu University Hospital, Fukuoka, Japan), Yoshihisa Fukushima, MD (St. Mary’s Hospital, Kurume, Japan), Ryu Matsuo, MD, PhD (Secretariat, Kyushu University, Fukuoka, Japan), and Kuniyuki Nakamura, MD, PhD (Kyushu University Hospital, Fukuoka, Japan).

***Event Adjudication Members:***

Ryu Matsuo, MD, PhD (Kyushu University), Jun Hata, MD, PhD (Kyushu University), Yasuhiro Kumai, MD, PhD (Hakujyuji Hospital), and Noriko Makihara, MD, PhD (Kyushu University).
